# Pseudonits or Hair Casts

**DOI:** 10.5826/dpc.1004a72

**Published:** 2020-10-26

**Authors:** Vania Lukoviek, Josep Malvehy, Susana Puig, Sebastian Podlipnik

**Affiliations:** 1 Department of Dermatology, University of Barcelona, Spain; 2 Department of Dermatology, University Hospital of Canarias, Tenerife, Spain

**Keywords:** pseudonits, nits, dermoscopy, polarized light dermoscopy

## Case Presentation

**Figure 1:**
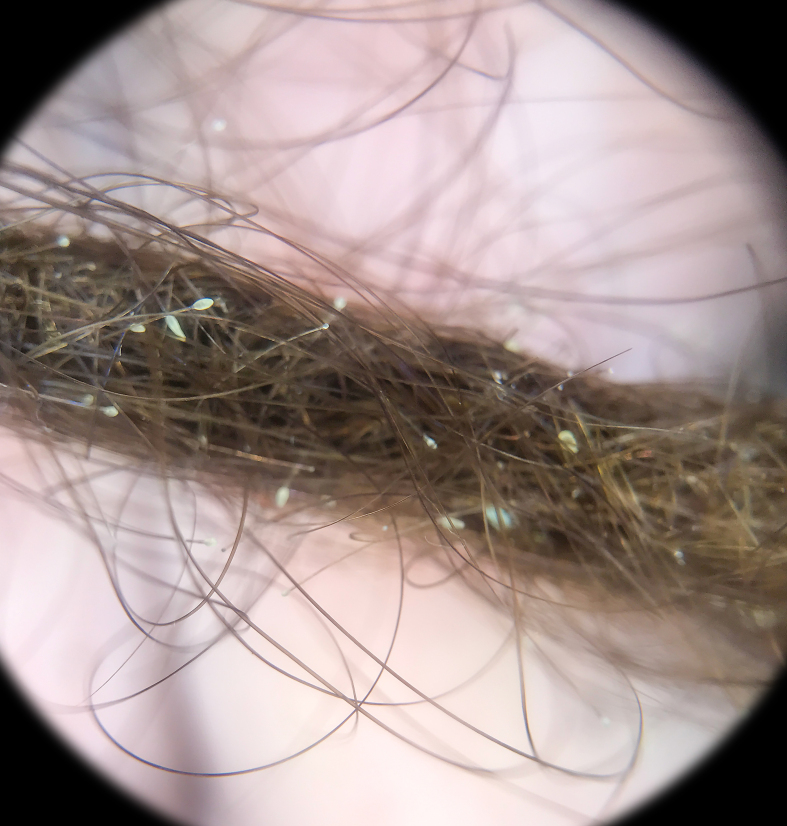
Trichoscopy reveals pseudonits.

A 34-year old female with an unremarkable medical history presented with a 3-month history of scalp pruritus. Physical examination revealed small whitish lesions resembling nits, abundant on one dreadlock. Trichoscopy showed many whitish, tubular and non-adherent structures on the hair shafts (Figure 1). There was no evidence of nits or lice on the rest of the scalp.

## Teaching Point

Pseudonits, hair casts, or keratin cysts are white objects that easily move along the hair. There are 2 types: (1) parakeratotic, associated with seborrheic dermatitis, psoriatic or excessive traction, and (2) peripilar, the pathogenesis of which is not clear but is more frequent in young women. Trichoscopy findings include whitish cylindrical structures around the hair shaft. Nits (main differential) present brown ovoid structure with a curved end (vital) or translucent structure with a planar end (empty). Pseudonits are frequently misdiagnosed as pediculosis capitis; trichoscopy, then, is essential for differentiation, avoiding patient anxiety and unnecessary treatments.
